# Matrix Rigidity Regulates Cancer Cell Growth by Modulating Cellular Metabolism and Protein Synthesis

**DOI:** 10.1371/journal.pone.0037231

**Published:** 2012-05-18

**Authors:** Robert W. Tilghman, Edik M. Blais, Catharine R. Cowan, Nicholas E. Sherman, Pablo R. Grigera, Erin D. Jeffery, Jay W. Fox, Brett R. Blackman, Daniel J. Tschumperlin, Jason A. Papin, J. Thomas Parsons

**Affiliations:** 1 Department of Microbiology, Immunology and Cancer Biology, University of Virginia, Charlottesville, Virginia, United States of America; 2 Department of Biomedical Engineering, University of Virginia, Charlottesville, Virginia, United States of America; 3 Molecular and Integrative Physiological Sciences, Harvard School of Public Health, Boston, Massachusetts, United States of America; University of Birmingham, United Kingdom

## Abstract

**Background:**

Tumor cells *in vivo* encounter diverse types of microenvironments both at the site of the primary tumor and at sites of distant metastases. Understanding how the various mechanical properties of these microenvironments affect the biology of tumor cells during disease progression is critical in identifying molecular targets for cancer therapy.

**Methodology/Principal Findings:**

This study uses flexible polyacrylamide gels as substrates for cell growth in conjunction with a novel proteomic approach to identify the properties of rigidity-dependent cancer cell lines that contribute to their differential growth on soft and rigid substrates. Compared to cells growing on more rigid/stiff substrates (>10,000 Pa), cells on soft substrates (150–300 Pa) exhibited a longer cell cycle, due predominantly to an extension of the G1 phase of the cell cycle, and were metabolically less active, showing decreased levels of intracellular ATP and a marked reduction in protein synthesis. Using stable isotope labeling of amino acids in culture (SILAC) and mass spectrometry, we measured the rates of protein synthesis of over 1200 cellular proteins under growth conditions on soft and rigid/stiff substrates. We identified cellular proteins whose syntheses were either preferentially inhibited or preserved on soft matrices. The former category included proteins that regulate cytoskeletal structures (e.g., tubulins) and glycolysis (e.g., phosphofructokinase-1), whereas the latter category included proteins that regulate key metabolic pathways required for survival, e.g., nicotinamide phosphoribosyltransferase, a regulator of the NAD salvage pathway.

**Conclusions/Significance:**

The cellular properties of rigidity-dependent cancer cells growing on soft matrices are reminiscent of the properties of dormant cancer cells, e.g., slow growth rate and reduced metabolism. We suggest that the use of relatively soft gels as cell culture substrates would allow molecular pathways to be studied under conditions that reflect the different mechanical environments encountered by cancer cells upon metastasis to distant sites.

## Introduction

Sensing the mechanical properties of the extracellular matrix (ECM) is a central mechanism for regulating the differentiation and proliferation of a multitude of cell types both *in vitro* and *in vivo*. Ample evidence implicates alterations in the signaling pathways that regulate the response of cells to microenvironmental cues as critical events in tumor initiation, progression, metastasis and perhaps tumor dormancy [1], [2]. In addition, the increase in tissue rigidity due to local accumulation of a dense, crosslinked collagen matrix is a hallmark of cancer progression in soft tissues and is the basis for detection of many types of tumors by physical palpation [3], [4].

Analysis of human cancer cell lines in cell culture is almost always performed using cells cultured on rigid plastic, or, less often, in Matrigel or soft agar, the mechanical properties of which are poorly defined and/or difficult to modulate. We have previously described a simple high-throughput method for culturing tumor cells on biologically relevant flexible substrates using ECM conjugated polyacrylamide (PA) gels that can span a stiffness range encompassing elastic moduli of 100 pascals ([Pa] or N/m2)–150,000 Pa [5]. In this assay we use a 96-well assay system that arrays PA gels of varying stiffness in user defined increments across the plate [6]. We have used this assay to assess how changes in the rigidity of the ECM modulate the biological properties of tumor cells, including growth, morphology, and migratory properties. The cancer cell lines tested were grouped into two categories based on their proliferation profiles: “rigidity dependent” lines exhibited increasing cell growth as extracellular rigidity increased, while “rigidity independent” lines grew equally well across the entire tested spectrum of matrix stiffness. Importantly, cells which grew poorly on soft gels exhibited decreased spreading and migration under these conditions and grew poorly when introduced into the soft tissue environment of the lung. The rigidity-dependent lung carcinoma line A549 responded to culture on soft gels by expressing the differentiated epithelial marker E-cadherin and decreasing the expression of the mesenchymal transcription factor Slug. Similarly, rigidity has also been found to modulate the epithelial-to-mesenchymal transition in normal epithelial cells [7]. These observations demonstrate that the mechanical properties of the matrix environment play a significant role in regulating the proliferation and the morphological properties of cancer cells, and that the “rigidity profile” is an intrinsic property of each cancer cell line.

Many cancer cell lines respond to less rigid microenvironments by proliferating more slowly; however, alterations in cellular metabolism due to changes in the rigidity of the microenvironment have not been well characterized. Cellular changes in metabolic processes such as protein synthesis may be especially relevant as tumor cells enter into a “dormant” state, primarily at sites of distant metastases where the cells have been introduced to a foreign microenvironment [8], [9]. In this study we investigated the properties of rigidity-dependent cancer cell lines that contribute to their differential growth on soft and rigid polyacrylamide substrates. Compared to cells growing on more rigid/stiff substrates, cells on soft substrates (150–300 Pa) exhibited a longer cell cycle, due predominantly to an extension of the G1 phase of the cell cycle, and were metabolically less active, showing decreased levels of intracellular ATP and a marked reduction in protein synthesis. To identify the proteins which exhibit differential synthesis rates when the cells are cultured on soft versus stiff gels, we used a recently developed application of stable isotope labeling of amino acids in culture (SILAC) and mass spectrometry to measure the rates of protein synthesis of over 1200 cellular proteins under conditions of growth on soft and rigid/stiff substrates [10]. Whereas overall rates of protein synthesis decrease markedly in rigidity-dependent cells grown on soft substrates, we identified cellular proteins whose syntheses were preferentially inhibited by culturing on soft substrates and proteins whose syntheses were relatively preserved when growing on soft matrices. The former category included proteins that regulate cytoskeletal structures (e.g., tubulin subunits) and glycolysis (e.g., phosphofructokinase P-1 and ATP synthase), whereas the latter category included proteins that regulate key metabolic pathways necessary for counteracting the damage from reactive oxygen species, such as aldo-keto reductase family members and nicotinamide phosphoribosyltransferase, a regulator of the NAD salvage pathway. The cellular properties of rigidity-dependent cancer cells growing on soft matrices are reminiscent of the properties of dormant cancer cells, i.e., slow growth rate and reduced metabolism. These data support the idea that the rigidity of the microenvironment can modulate tumor cell proliferation by modulating fundamental cellular processes. Further, the soft plate technology may provide a unique platform for the study of cancer cells undergoing dynamic regulation of proliferation in response to changes in the mechanical environment, thus providing insights into how microenvironmental changes modulate cancer cell growth in experimental animal models and in patients.

## Results

### Cell cycle progression of rigidity-dependent cells on soft gels

We have previously shown that a panel of cancer cell lines can be segregated based upon the ability to proliferate on soft (<1000 Pa) collagen-coated gels. A549 cells (lung carcinoma) and MDA-MB-231 cells (breast carcinoma) are classified as “rigidity-dependent” and exhibit doubling times that are at least 2 fold longer on soft versus stiff matrices. In contrast, the “rigidity-independent” mPanc96 cells (pancreatic carcinoma) grow equally well on soft compared to stiff matrices and have similar doubling times under these conditions ([5], unpublished observations).

**Figure 1:**
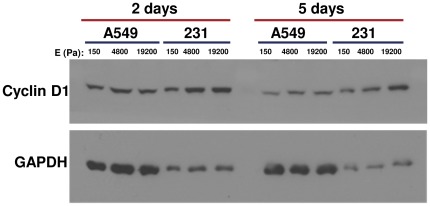
Cyclin D1 expression in rigidity-dependent cells growing on soft and stiff gels. A549 cells and MDA-MB-231 cells were cultured on 150 Pa, 4800 Pa, or 19200 Pa polyacrylamide gels for 2 or 5 days. Cells were lysed and analyzed by western blot for the expression of cyclin D1 (top panel). The expression of GAPDH was analyzed as a loading control (bottom panel). The blot is representative of three experiments.

To understand the molecular basis for the slow growth of rigidity-dependent cancer cell lines on soft matrices, we measured key regulators of cell cycle progression as well as the time required for cells to transverse the cell cycle on soft or stiff substrates. Cyclin D1 is critical for cells to enter the cell cycle, and the loss of cyclin D1 expression is a marker for cells that have exited the cell cycle and are quiescent [11]. Additionally, cyclin D1 expression has been shown to be regulated by matrix rigidity through the FAK-dependent activation of Rac in untransformed cells [12]. The expression of cyclin D1 in rigidity-dependent cancer cells was measured to determine whether the cells exit the cell cycle when cultured on soft gels. Rigidity-dependent A549 or MDA-MB-231 cells were cultured on 150 Pa, 4800 Pa, or 19200 Pa gels for 2 or 5 days, and cyclin D1 expression was measured by western blot. Both cell lines still expressed cyclin D1 even when cultured on the soft (150 Pa) gels (Figure 1). These data indicate that rigidity-dependent cells do not exit the cell cycle, even on soft gels where the cells exhibit slower growth.

**Figure 2:**
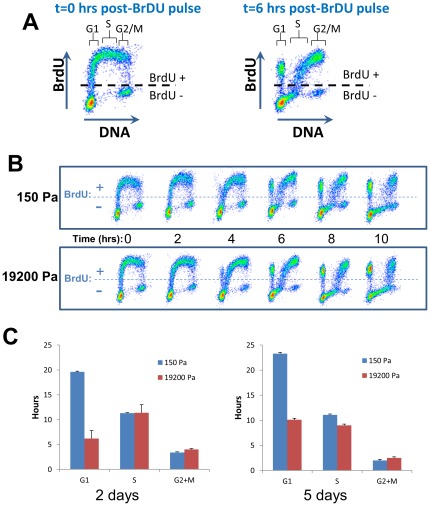
BrdU pulse-chase of cell cycle progression. A549 cells were pulsed with BrdU for 30 minutes following growth on soft or stiff gels for 2 days. **A.** Labeling cells with BrDU for cell cycle analysis. Cells are pulsed with BrDU for 30 min, and the BrDU-positive population is followed over time as it transitions through the phases of the cell cycle. **B.** Scatter plot histograms of BrdU-labeled cells on soft (top panel) or stiff (bottom panel) gels, stained for DNA content (X-axis) and BrdU (Y-axis). The times indicated are the times, in hours, after the BrdU pulse. **C.** Cell cycle progression analysis was performed on the scatter plot histograms from the cells grown on gels for 2 days (left) or 5 days (right).

Since rigidity-dependent A549 cells do not exit the cell cycle when plated on soft gels, and only ∼5% of the cells undergo apoptosis under these conditions [5], we hypothesized that these cells are progressing through the cell cycle more slowly than when they are growing on stiff substrates. To examine the rate of progression through specific phases of the cell cycle, the rigidity-dependent lung carcinoma line A549 was cultured on soft (150 Pa) or stiff (19200 Pa) gels for two or five days and then pulsed for 30 minutes with the nucleotide analog bromodeoxyuridine (BrdU) to label the population of cells undergoing DNA synthesis. Time spent in each phase of the cell cycle was determined by FACS to track the BrdU-positive population as the cells progressed through the S and G2 phases and accumulated in the G1 phase [13]. As shown in Figure 2, cells on soft gels progressed more slowly through the G1 phase of the cell cycle compared to the same cells growing on stiff matrices (Figure 2), consistent with a global decrease in cellular metabolism or anabolic processes that affect the cellular “growth” stage of the cell cycle. Because the cell cycle profiles are similar after 2 and 5 days on soft gels, it is likely that this represents a steady-state measurement, as opposed to the possibility that cell growth is gradually slowing down over time. Based on the calculated length of the cell cycle for cells growing on soft versus stiff gels, after 5 days the ratio of cells on soft versus stiff matrices would be 1∶4.3. The same ratio of growth on soft versus stiff gels (1∶4.3) was observed by manually counting the number of cells present at five days, thereby validating the BrdU pulse-chase calculations.

### Cellular metabolism in rigidity-dependent cells cultured on soft gels

**Figure 3:**
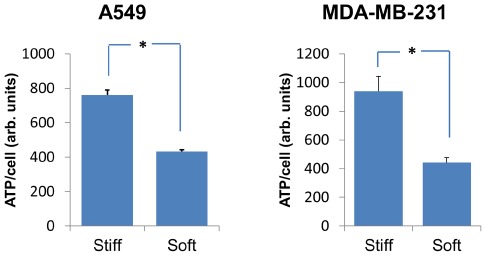
ATP levels in rigidity-dependent cells cultured on soft or stiff gels. ATP levels were measured in A549 cells (left) or MDA-MB-231 cells (right) following culture on polyacrylamide gels for 2 days. Data represents the average of two experiments performed in triplicate ± S.E., of cells on soft (300 Pa) or stiff (19200 Pa) gels. Total ATP levels were normalized to cell numbers. * p<0.05.

The G1 phase of the cell cycle relies heavily upon cellular metabolic events and is critical for the synthesis of structural proteins and enzymes that contribute to the generation of new organelles and the overall growth of the cell. Because the G1 phase of the cell cycle was prolonged in rigidity-dependent cells grown on soft gels, we hypothesized that there may be metabolic changes under conditions of growth of soft substrates. Culturing the rigidity-dependent A549 or MDA-MB-231 cells on soft gels for 2 days resulted in an approximately 50% decrease in ATP levels compared with cells growing on stiff gels (Figure 3). Like the lengthening of the G1 phase, the lower levels of cellular ATP are consistent with a decrease in cellular metabolism when rigidity-dependent cells are cultured on soft gels.

### Effect of rigidity on protein synthesis in cells cultured on soft gels

**Figure 4:**
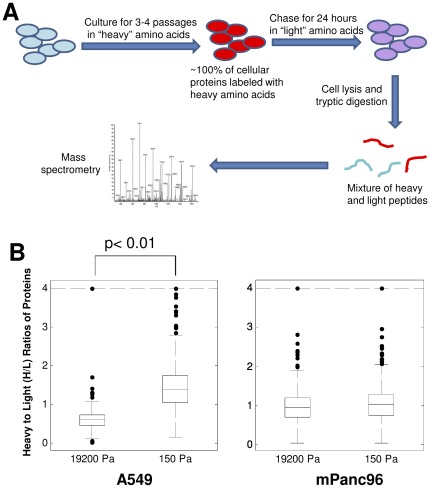
Protein synthesis is decreased in rigidity-dependent cells cultured on soft gels. A549 cells were subjected to SILAC analysis to determine rates of protein synthesis on soft or stiff gels. **A.** Overview of the SILAC procedure. A549 cells were cultured on soft or stiff gels for 4 days in the presence of “heavy” media, followed by a 24-hour incubation with “light” media. The cells were lysed, cellular proteins were digested with trypsin, and the resulting peptides were analyzed by mass spectrometry. **B.** Boxplots of heavy to light (H/L) ratios of proteins from A549 cells (left) or mPanc96 cells (right) grown on stiff (19200 Pa) or soft (150 Pa) substrates. H/L ratio distributions are significantly different between stiff and soft for A549 cells but not for mPanc96 cells using two-tailed unpaired t-tests. The boxes contain the data between the 25 and 75 percentile, and the line within the box indicates the median. The dashed line at the top of the graph marks the upper limit, above which the outliers were truncated.

Because of the prolonged G1 phase and the decrease in ATP levels in cells cultured on soft gels, we determined whether matrix rigidity may regulate net protein synthesis in rigidity-dependent cells. To assess the changes in global protein synthesis, and to identify proteins that might be differentially synthesized under soft versus stiff conditions, we used a recently developed application of stable isotope labeling of amino acids in cell culture (SILAC) in conjunction with mass spectrometry [10]. Rigidity-dependent A549 cells and rigidity-independent mPanc96 cells were grown in SILAC media for two generations on plastic tissue culture dishes to fully label the proteome with “heavy” amino acids (Figure 4A). Cells were then plated on either soft or stiff gels and further cultured in “heavy” amino acids for an additional 4 days. Cells were then incubated for 24 hours (“pulse”) in “light” media (normal tissue culture media), and cellular proteins were isolated from each of the samples and resolved on SDS-PAGE. Proteins present in soft and stiff samples were analyzed by taking ten gel slices from each protein track and subjecting each to digestion with trypsin. Net protein synthesis during the 24 hour “pulse” was measured by determining the ratio of heavy to light peptides by mass spectrometry for proteins in the soft and stiff samples. A preliminary experiment with A549 cells cultured on plastic yielded heavy to light ratios (H/L ratios) of peptides that were similar to those of A549 cells cultured on stiff gels (0.6246±0.2326 for plastic vs. 0.5482±0.1785 for stiff gels). Figure 4B shows boxplots for H/L ratios of proteins identified for A549 and mPanc96 cells on soft and stiff gels using SILAC. The average heavy/light ratio of peptides in the A549 cells on soft gels was significantly higher (p<0.05) than in the cells on stiff gels, indicating that net protein synthesis was decreased in cells on soft gels, (i.e., fewer light amino acids were incorporated during the pulse in cell growing on soft substrates compared to stiff substrates). In contrast, the heavy/light ratios of peptides in mPanc96 cells were not significantly different between cells growing on soft or stiff gels (p>0.05) (Figure 4B).

**Figure 5:**
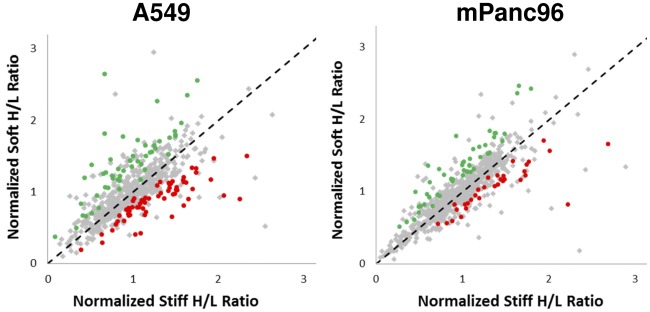
Identification of proteins that are differentially regulated by rigidity. H/L ratios of proteins identified in both stiff and soft samples plotted against each other for A549 cells (left) and mPanc96 cells (right). H/L ratios from Figure 4 were quantile normalized and t-tests were performed using estimated variability (see Methods) to identify individual proteins with relatively different synthesis rates between stiff and soft samples. Proteins that are synthesized faster (relatively lower H/L ratio and p-value<0.05) in soft samples (compared to stiff samples) are shown in red, and proteins that are synthesized slower in soft samples are shown in green.

The mass spectrometry analysis identified 631 unique proteins in the A549 cells and 729 unique proteins in the mPanc96 cells (Tables S1 and S2). Relative rates of synthesis (H/L ratios) for individual proteins were then compared between cells grown in stiff and soft conditions to determine proteins that are differentially “expressed” or translated. Since the distributions of H/L ratios differed between stiff and soft experiments, H/L ratios and estimated variability were normalized independently for A549 and mPanc96 samples (see Methods), and we identified proteins with significantly different H/L ratios (p<0.05) using t-tests. Figure 5 shows the scatterplots of normalized H/L ratios between samples growth on stiff and soft substrates. Proteins that have a significantly smaller H/L ratio (preserved synthesis) in soft compared to stiff gels are indicated in red and proteins that have a significantly higher H/L ratio (slower synthesis) in soft than stiff gels are indicated in green. The dashed line indicates the expected H/L ratio under the null hypothesis that proteins maintain the same H/L ratio relative to the mean.

From the proteins identified by mass spectroscopy, we separated out the proteins that exhibited significantly different H/L ratios (p<0.05) between cells cultured on stiff and soft gels (Table S3 for A549 cells; Table S4 for mPanc96 cells). In A549 cells, several proteins with common biological functions clustered in the category of slower synthesis on soft gels (Table 1). These included proteins involved in protein synthesis such as ribosomal proteins and elongation factors. Subunits of microtubules (tubulins) also clustered in the category of slower synthesis on soft gels. Interestingly, two proteins with crucial roles in ATP production also displayed slower synthesis rates on soft gels: 6-phosphofructokinase type C (PFKP-1) and a subunit of ATP synthase. These data suggest that when A549 cells are cultured in a soft microenvironment, a subset of proteins - including those involved in translation and microtubule dynamics - is synthesized more slowly relative to the average synthesis rate than when cultured on stiff gels.

**Table 1:** Proteins in A549 cells identified by mass spectrometry whose syntheses are most sensitive to changes in rigidity.

Category	Protein	H/L Difference	P value
**Protein Synthesis**	39S ribosomal protein L19, mitochondrial	−0.84825	4.92E-06
	60S ribosomal protein L3 isoform b	−0.83025	0.001151
	Isoform Cytoplasmic of Lysyl-tRNA synthetase	−0.69964	7.34E-05
	Elongation factor 1-gamma	−0.46961	4.31E-05
	Asparaginyl-tRNA synthetase, cytoplasmic	−0.45776	0.008219
	Eukaryotic translation initiation factor 2 subunit 1	−0.39179	0.009855
	Elongation factor 2	−0.24762	0.035289
	Elongation factor 1-beta	−0.24342	0.039255
	cDNA FLJ55750, highly similar to Eukaryotic translation initiation factor 3 subunit 8	−0.17977	0.024772
	Eukaryotic translation initiation factor 6	−0.15971	0.027737
**Microtubules**	cDNA FLJ32385 fis, clone SKMUS1000110, highly similar to Tubulin alpha-8 chain	−0.3963	0.003499
	Tubulin beta-3 chain	−0.29527	0.012761
	Tubulin beta-2A chain	−0.27029	2.36E-05
	cDNA FLJ58687, highly similar to Tubulin alpha-4 chain	−0.26169	0.049913
	Tubulin, beta	−0.23846	0.009491
	cDNA FLJ53012, highly similar to Tubulin beta-7 chain	−0.12995	0.001608
**ATP Production**	ATP synthase subunit beta, mitochondrial	−1.05954	5.18E-06
	6-phosphofructokinase type C	−0.88302	2.44E-05

The table lists proteins that exhibited higher heavy/light ratios (and therefore, lower synthesis) on soft gels compared to the mean.

Conversely, proteins that had higher synthesis rates than the bulk of the cellular proteins when plated on soft gels included members of the aldo-keto reductase family, which are involved in protection against reactive oxygen species, and nicotinamide phosphoribosyltransferase, which is important for maintaining physiological levels of the nicotinamide cofactors (i.e., NADPH) that are important for these redox reactions (Table 2). Additionally, proteins with roles in endocytosis and vesicle trafficking, specifically annexins and Rab proteins, were also found to be conserved in A549 cells cultured on soft gels. The data in this table suggest that when A549 cells are cultured in a soft microenvironment, the synthesis of proteins that are important for the regulation of reactive oxygen species and membrane dynamics is preserved.

**Table 2:** Proteins in A549 cells identified by mass spectrometry whose syntheses are least sensitive to changes in rigidity.

Category	Protein	H/L Difference	P value
**Antioxidants**	Aldo-keto reductase family 1 member C1	1.119452	5.86E-11
	Aldo-keto reductase family 1 member B10	0.830564	1.77E-06
	Aldo-keto reductase family 1 member C2	0.691516	1.66E-05
	Aldo-keto reductase family 1 member C3	0.458775	0.004641
	Epoxide hydrolase 1	0.321919	0.001640
	Aldehyde dehydrogenase, dimeric NADP-preferring	0.267702	0.027041
	Peroxiredoxin-4	0.23855	0.036688
**Vesicle Trafficking**	Ras-related protein Rab-27B	0.637138	6.96E-07
	Annexin A3	0.399658	0.013510
	annexin A4	0.317661	0.014001
	Annexin A1	0.267954	0.012983
	Ras-related protein Rab-5C	0.25913	0.017720
	Ras-related protein Rab-21	0.187404	0.011429
**NAD Production**	Nicotinamide phosphoribosyltransferase	0.767791	3.72E-08
	Kynureninase	0.294088	0.000371

The table lists proteins that exhibited lower heavy/light ratios (and therefore, higher net synthesis) on soft gels compared to the mean.

Similar to A549 cells cultured on soft gels, several ribosomal proteins were also synthesized more slowly in mPanc96 cells that were cultured on soft gels (Table 3). However, unlike the A549 cells, several proteins with roles in translation were also found to have higher synthesis rates in mPanc96 cells when cultured on soft gels (Table 4). Additionally, while the A549 cells showed proteins involved in vesicle trafficking and oxidative stress pathways as having preserved synthesis rates on soft gels, proteins involved in these processes, such as coatomer subunits and superoxide dismutase, respectively, were synthesized more slowly in mPanc96 cells cultured on soft gels (Table 3), suggesting that these processes may be downregulated when mPanc96 cells are growing in soft microenvironments. Interestingly, several proteins that are involved in the regulation of cell-matrix adhesion and actin dynamics, including the Rac family member RhoG, fascin, and inverted formin-2, were found to have higher rates of synthesis when mPanc96 cells are cultured on soft gels (Table 4), in addition to two proteins (citrate synthase and malate dehydrogenase) that have crucial roles in the citric acid cycle, an important metabolic process for the production of ATP and amino acid precursors. These data suggest that when the rigidity-independent cell line mPanc96 is cultured in a soft microenvironment, there is a decrease in the synthesis of proteins involved in vesicle trafficking and oxidative stress pathways, while there is an increase in the synthesis of proteins involved in actin dynamics and the citric acid cycle.

**Table 3:** Proteins in mPanc96 cells identified by mass spectrometry whose rates of syntheses decrease when cultured on soft gels.

Category	Protein	H/L Difference	P value
**Protein Synthesis**	60S ribosomal protein L3 isoform b	−0.50342	0.003728
	40S ribosomal protein S17	−0.42207	0.006099
	40S ribosomal protein S10	−0.35175	0.008133
	cDNA FLJ35809 fis, clone TESTI2006016, highly similar to Eukaryotic translation initiation factor 3 subunit 3	−0.29715	0.019452
	40S ribosomal protein S4, X isoform	−0.2837	0.028561
	60S acidic ribosomal protein P0	−0.26139	0.019264
	60S ribosomal protein L13a	−0.22628	0.043236
	Isoform Complexed of Arginyl-tRNA synthetase, cytoplasmic	−0.18043	0.013539
**Vesicle Trafficking**	Coatomer subunit zeta-1	−0.5522	0.008988
	Isoform 1 of Coatomer subunit alpha	−0.52696	0.011334
	Vesicle-trafficking protein SEC22b	−0.46288	0.004961
	Cytoplasmic dynein 1 heavy chain 1	−0.23752	0.031111
**Antioxidants**	Glutathion reductase delta8+9 alternative splicing variant	−0.81552	9.61E-05
	Peroxiredoxin-1	−0.3869	0.000954
	Superoxide dismutase [Cu-Zn]	−0.10054	0.033937

The table lists proteins that exhibited higher heavy/light ratios (and therefore, lower synthesis) on soft gels compared to the mean.

**Table 4:** Proteins in mPanc96 cells identified by mass spectrometry whose rate of syntheses increase when cultured on soft gels.

Category	Protein	H/L Difference	P value
**Protein Synthesis**	Translational activator GCN1	0.326805	0.014649
	40S ribosomal protein S5	0.247387	0.026861
	IARS protein	0.10338	0.001431
**Actin Dynamics**	Rho-related GTP-binding protein RhoG	0.269265	0.001418
	Fascin	0.239673	0.020165
	Isoform 2 of Inverted formin-2	0.201525	0.027210
	Isoform 1 of Myosin-14	0.176649	0.032630
**Citric Acid Cycle**	Citrate synthase, mitochondrial	0.455628	8.05E-05
	Malate dehydrogenase, mitochondrial	0.313488	0.028990

The table lists proteins that exhibited lower heavy/light ratios (and therefore, higher net synthesis) on soft gels compared to the mean.

**Figure 6:**
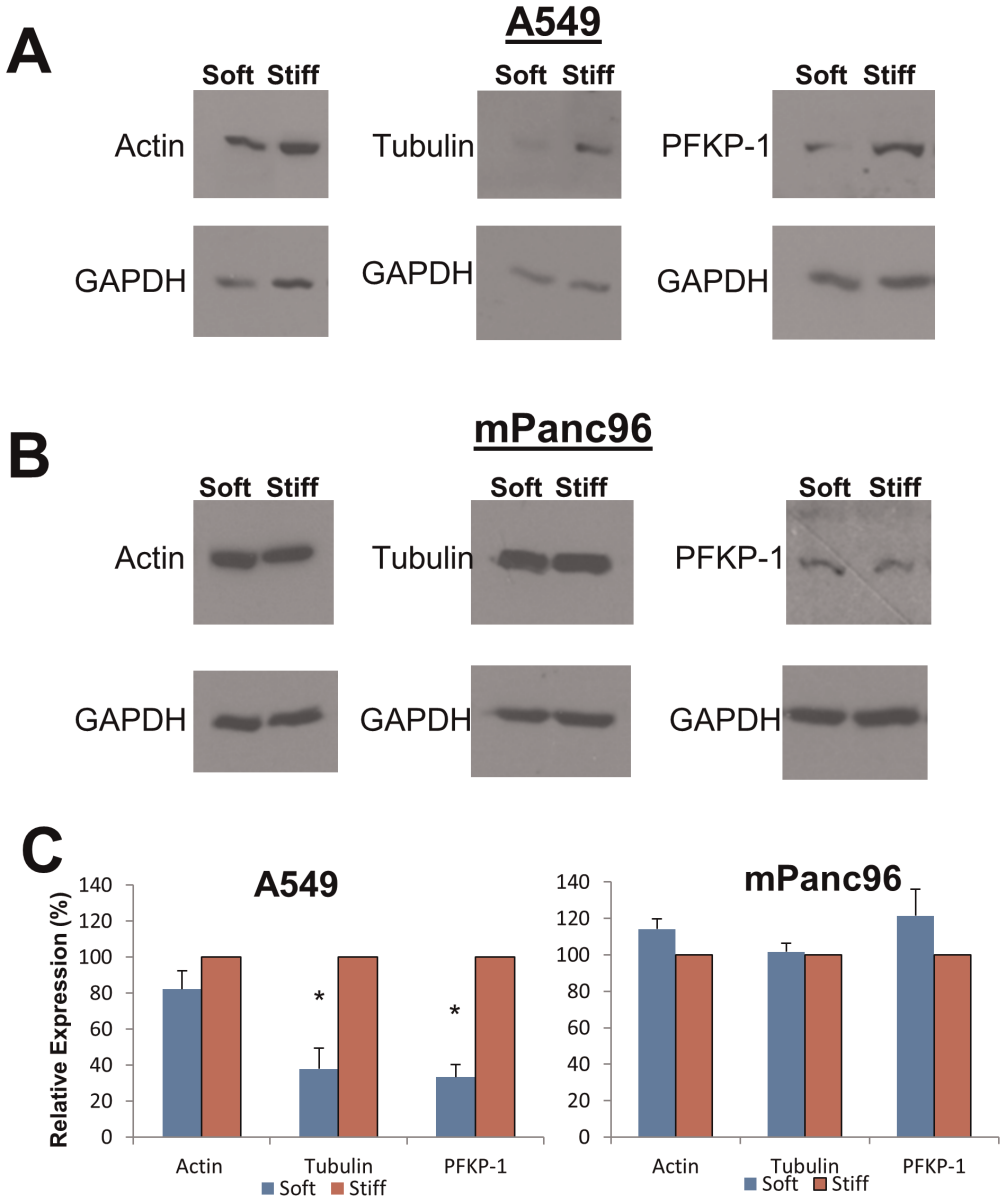
Cellular levels of proteins identified by mass spectrometry. **A.** The levels of actin, tubulin, and phosphofructokinase-1 in A549 cells cultured on soft (150 Pa) or stiff (19200 Pa) gels were analyzed by western blot. Bottom panels show levels of GAPDH as a loading control. **B.** The levels of actin, tubulin, and PFKP-1 in mPanc96 cells cultured on soft or stiff gels as analyzed by western blot. Bottom panels show levels of GAPDH in cell lysates as a loading control. **C.** Quantitation of western blot results in (A) and (B). The levels of each protein were normalized to the amount of GAPDH in each sample. The levels of protein in samples prepared from stiff gels were set to 100%, and the levels of protein in the soft samples are displayed as percentage expression of these samples. Results show the mean ± S.E. of at least three experiments. * p<0.05 when compared to the level of actin expression.

Because these data reflect only the synthesis of the proteins, and the overall levels of the proteins are determined by the balance of synthesis and degradation, we sought to test whether this alteration of protein synthesis was actually affecting the cellular levels of these proteins. Lysates from A549 and mPanc96 cells cultured on soft or stiff gels were subjected to western blot for actin, a protein that had showed no significant changes in relative synthesis when cultured on soft gels, and two proteins that showed high sensitivity to rigidity, namely, tubulin and phosphofructokinase-1. The levels of the proteins were normalized to GAPDH as a loading control, because there was not a significant difference in the relative synthesis of GAPDH between soft and stiff gels (p = 0.55, see Table S1, line 275). As shown in Figure 6, actin was expressed at similar levels when cultured on soft or stiff gels, while the levels of tubulin and PFKP-1 were lower on the soft gels in A549 cells, suggesting that the lower rate of synthesis of these proteins observed in the SILAC data is reflected by lower levels of these proteins in cells. In contrast, mPanc96 cells showed no changes in tubulin or PFKP-1 levels when cultured on soft gels. These data indicate that, at least for the proteins examined (tubulin and PFKP-1), a slower rate of synthesis corresponds to lower levels of cellular protein.

## Discussion

In this paper, we demonstrate that the rigidity of the extracellular matrix regulates cellular metabolism and protein synthesis in cancer cells. The rigidity-dependent cancer cell lines A549 and MDA-MB-231 sustain the expression of cyclin D1 when cultured in serum on polyacrylamide gels that have mechanical properties similar to that of soft tissue such as lung or breast. In spite of cyclin D1 expression, A549 cells, when cultured on soft gels, show a lengthening of the G1 phase of the cell cycle, suggesting that there may be defects in the synthesis of the structural and enzymatic components necessary for the transition into S phase. Accordingly, the rigidity-dependent cell lines show lower levels of cellular ATP levels when cultured on soft gels. Additionally, protein synthesis in A549 cells is slower under this condition, with the synthesis of specific structural proteins and glycolytic enzymes such as tubulin, phosphofructokinase, and ATP synthase especially sensitive to the decrease in extracellular rigidity. Proteins that were less sensitive to the change in rigidity included enzymes such as aldo-keto reductase and nicotinamide phosphoribosyltransferase that are involved in the metabolism of ROS products.

Matrix rigidity has been suggested to be a mechanism underlying the tissue tropism of cancer metastases, because growth of carcinoma cell lines on soft or stiff gels correlates with their ability to grow in soft or stiff tissues [5], [14]. However, the mechanism(s) that regulate the growth of cancer cells in a tissue-specific manner are undoubtedly complicated. Our observations suggest that one way in which rigidity-dependent cancer cells may respond to a less than favorable tissue-matrix environment is to decrease their metabolic state, which offers one potential mechanism for the observed tissue tropism of tumor cells, and perhaps tissue-dependent tumor dormancy.

Tumor dormancy is defined as a stage in cancer progression in which residual disease is present but is asymptomatic [15]. Dormant cancer cells can reside undetected at sites distant from the primary tumor, pending subsequent growth and clinical recurrence [16]. Recent studies support a model whereby cancer cells from the primary tumor will disseminate early during disease progression, so that by the time the primary tumor is detected, there may be dormant cancer cells already residing at distant sites [17]. These cells are either temporarily nonproliferative, or there is a balance between proliferation and cell death, or the immune system is keeping the cell numbers in check. Interestingly the latter possibility has come under scrutiny because of studies showing a lack of tumor recurrence in cancer patients that have undergone immunosuppressive therapy [18].

Recent studies have implicated the microenvironment as a key regulator of tumor cell dormancy and recurrence. Specifically, the properties of the extracellular matrix at sites of metastasis may play important roles in determining the state of disseminated tumor cells [9]. However, mechanistic insight as to how dormancy occurs is scarce, since dormant cancer cells are difficult to detect and study in vivo, and there is a paucity of in vitro models for tumor cell dormancy. Until recently, such models have been limited to cells grown on chorioallantoic membranes (CAMs) or on 3D matrix derived from the Engelbreth-Holm-Swarm tumor (EHS matrix, or Matrigel) [19], [20]. Matrigel is a mixture of laminin, collagen IV, and nidogen, in addition to a variety of proteases and growth factors such as TGFβ, FGF, EGF, PDGF, and IGF [21]. Because it is generated from a tumor, the specific composition of Matrigel is not well defined, and it may vary from batch to batch, producing variability in experimental results [22]. While the mechanical properties of Matrigel have been analyzed, they also are subject to variability because its polymerization is affected by its composition and other experimental factors such as temperature [23]. In addition, currently the only way to alter the mechanical properties of Matrigel is to add additional matrix components such as collagen I [24], which may also complicate the interpretation of experimental results.

Recently, in vitro models for tumor dormancy have expanded to include the use of polyacrylamide gels as growth substrates [25]. Unlike Matrigel, polyacrylamide is a stable, homogeneous polymer, and its mechanical properties are not sensitive to changes in temperature [26]. Its rigidity is easily tunable by modulating the amount of bis crosslinker without affecting its ability to bind ECM molecules to its surface [6]. Polyacrylamide hydrogels can encompass a spectrum of elastic moduli that includes a range of human tissues from fat to skeletal muscle [27], and a variety of ECM molecules can be conjugated to its surface, including collagen, fibronectin, and unpolymerized Matrigel. In addition, a high-throughput system was recently developed to enable the screening of small molecule inhibitors with cells cultured on polyacrylamide gels spanning a range of elastic moduli [5], [6]. In this study, we have used polyacrylamide gels to mimic the mechanical properties of the soft tissue that cancer cells commonly encounter during metastasis, such as the lung, liver, and bone marrow.

We have shown that the growth of certain cancer cell lines slows when cultured on soft gels [5]. In this study, we provide evidence that cellular metabolic events are key regulators promoting the slow growth of rigidity-dependent cells on soft matrices. Little is known about the regulation of cellular metabolism by cell adhesion to the extracellular matrix. Recent evidence shows that in some cancer cells, autophagy is linked to metabolic changes [28]. However we observed no changes in autophagy in cells cultured on soft gels as measured by the conversion of the autophagocytic marker LC3-I to LC3-II (data not shown). In addition, a comparison of the gene expression profiles of 559 genes encoding proteins in the SILAC data set from A549 cells cultured on soft and stiff gels showed no correlation between changes in protein synthesis and mRNA levels (data not shown). These data are in agreement with early studies conducted by Penman and colleagues in which they showed that fibroblasts in suspension exhibited a decrease in protein synthesis that was rapidly recovered when the cells were re-plated onto plastic and that these changes were unrelated to changes in mRNA synthesis. [29], [30], [31]. The mechanism(s) that leads to the halt in protein synthesis is unclear; however, it is interesting to note that several ribosomal proteins and elongation factors are synthesized slower when A549 cells are cultured on soft gels. It is also interesting that several of the proteins that are sensitive to changes in rigidity are proteins involved in the generation of ATP – a process that determines the metabolic state of the cell and could affect protein synthesis. One of these proteins, phosphofructokinase P-1 (PFKP1, or 6-phosphofructokinase type C) is a key regulator of glucose metabolism by catalyzing the first “committed” step in glycolysis, the conversion of fructose 6-phosphate to fructose 1,6-biphosphate [32]–[33]. Levels of phosphofructokinase have been shown to be regulated by insulin and nutrients [34], and its levels are decreased in a rat model of diabetes [35], suggesting that it is an important regulatory point in glucose metabolism. This function is in agreement with our observation that ATP levels are lower in cells cultured on soft gels, although it remains to be seen as to whether these two events are causally linked.

It is also interesting to note that the synthesis of other metabolic enzymes such as aldo-keto reductase and nicotinamide phosphoribosyltransferase (Nampt) are maintained when A549 cells are moved from stiff to soft environments. Aldo-keto reductase is involved in the detoxification of ROS products and xenobiotic molecules [36], which could be critical for the survival of cancer cells, perhaps even in a “dormant” state, due to the high levels of ROS produced by cancer cells [37], [38]. Nampt is the rate-limiting step in the salvage pathway to generate NAD from nicotinamide [39], which is important for production of NADPH, an important cofactor for aldo-keto reductases and other reactions that play vital roles in the protection of cells from the toxicity of ROS. Therefore, the conservation of proteins such as aldo-keto reductase and Nampt in cells cultured on soft gels suggests that these proteins may be critical for survival even in soft microenvironments where the cells are proliferating more slowly. This property is in line with recent studies demonstrating that targeting enzymes critical for protection against oxidative stress has therapeutic potential [40].

In summary, our observations are consistent with a “slowing down” in the growth and metabolism of cancer cells as they move from a stiff to a soft environment. Future studies will be necessary to determine if these metabolic changes occur in vivo when cancer cells are placed in soft tissues to mimic the dissemination of the disease.

## Materials and Methods

### Cell lines and antibodies

The lung cancer cell line A549, the breast cancer cell line MDA-MB-231, and the pancreatic cancer cell line mPanc96 were obtained from the ATCC. All cells were routinely cultured in RPMI supplemented with 10% fetal bovine serum (FBS) and penicillin/streptomycin. Antibodies to cyclin D1 and phosphofructokinase-1 were purchased from Abcam, the actin antibody was purchased from Sigma, and the FITC-BrdU antibody was from Invitrogen.

### Polyacrylamide substrates

Flexible polyacrylamide substrates were generated on glass coverslips and adapted for cell culture using the method of Pelham and Wang [24]. Polyacrylamide gels contained 3% (150 Pa) or 7% acrylamide (4800 and 19200 Pa), and 0.04% (150 Pa), 0.05% (4800 Pa), or 0.24% (19200 Pa) bisacrylamide. The gels were polymerized on acid-washed, silanated, and glutaraldehyde-treated 22 mm glass coverslips. Each gel was placed in a well of a 6-well dish and activated using the heterobifunctional crosslinker sulfosuccinimidyl-6(4′-azido-2′-nitrophenylamino) hexanoate (Sulfo-SANPAH) followed by coating with collagen I (10 µg/ml) for four hours at room temperature or overnight at 4°C. The gels were soaked in the appropriate growth media at 37°C for at least 20 minutes prior to the addition of cells. Polyacrylamide coated 96-well plates were fabricated and prepared for cell culture as previously described [6].

### Cell cycle analysis

A549 cells were cultured on 150 Pa or 19200 Pa gels for 3 or 5 days. BrdU was added to the cells for 30 minutes; cells were washed twice with PBS and incubated for 2–10 hours in fresh growth media. Labeled cells were collected by trypsinization, washed twice with PBS, and resuspended in 0.5 ml PBS. The cells were fixed overnight in 4 ml of 70% ethanol. The fixed cells were collected by centrifugation, and rehydrated in 5 ml PBS for 10 minutes. The cells were again collected by centrifugation and DNA was denatured for 30 minutes in 0.3 ml of a solution of 0.2 mg/ml pepsin in 2M hydrochloric acid. Following denaturation, the acid was neutralized with 1 ml of a solution of 3.8% sodium tetraborate (pH 8.5). The cells were then washed once with 1 ml of FACS buffer (2% BSA, 0.1% Triton-X 100, 0.1% sodium azide in PBS) and incubated with anti-BrdU antibody (diluted 1∶8 in FACS buffer) for 1 hour at room temperature. The cells were washed once with FACS buffer and resuspended in PI staining solution (0.1% Triton X 100, 0.1% RNAase cocktail [Ambion], 0.02 mg/ml PI in PBS). The stained cells were analyzed by flow cytometry on a BD FACSCalibur Benchtop Analyzer. Scatter charts were analyzed with FlowJo v8.8.6. The length of the S phase, G2+M phase, and the potential doubling time were calculated by measuring the percentage of the BrDU-positive population in each phase for each timepoint, and the length of the G1 phase was determined by subtracting the lengths of the S and G2+M phases from the potential doubling time, as previously described [13].

### ATP assay

A549 or MDA-MB-231 cells were cultured in 96-well plates on polyacrylamide gels with an elastic modulus range of 300 Pa (“soft”) to 19200 Pa (“stiff”) for two days. The cells were lysed, and cellular ATP levels were measured using the ATPlite assay (Perkin Elmer), as per the manufacturer's instructions. The number of cells in each modulus was determined by using the CyQuant cell proliferation assay on cells in parallel wells, and the cellular ATP was normalized to cell number for each condition.

### Western Blotting of lysates from cells on gels

Western blotting was performed by directly lysing the cells on the gels with sample buffer and scraping the gels from the coverslips, followed by removal of the gel from the lysates by centrifugation through glass wool as previously described [5]. The proteins in the lysates were separated by SDS-PAGE and transferred to a nitrocellulose membrane. Equal amounts of protein were confirmed in each lane on the gel by Ponceau staining.

### SILAC of A549 and MPanc96 cells

A549 or mPanc96 cells were grown for two passages in SILAC media (Lysine and Arginine replaced with Lys 13C6 and Arg 13C6, respectively) to incorporate the heavy isotopes into the cellular proteins. The labeled cells were then cultured on soft (150 Pa) or stiff (19200 Pa) gels for 4 days In the presence of heavy amino acids. On the fourth day, the cells were washed twice in PBS, and incubated for 24 hours in unlabeled (“light”) media. The cells were then collected by trypsinization, counted, and lysed in sample buffer. Lysates were separated by SDS-PAGE, and protein bands were cut from the gel and digested with trypsin. The resulting peptides were analyzed and identified by mass spectrometry [41], and for each individual protein the number of heavy (H) or light (L) peptides was determined. H/L ratios and variability percentages for each protein were averaged and weighted by spectral area across 10 protein bands. Since overall distributions of H/L ratios may differ between samples similar to mRNA expression arrays, all protein H/L ratios identified in both stiff and soft were normalized using quantile normalization in MATLAB (Mathworks). Welch's t-tests were performed to determine significant differences between proteins in samples grown on stiff and soft substrates using: average H/L ratio (quantile normalized), variance (based on mass spec called variability percentage), and estimated degrees of freedom (using log2 of total spectral area as an estimated number of samples, see Tables S1 and S2 for full list of variables). Proteins that were identified as statistically significant are summarized in Tables S3 and S4, with differences between stiff and soft H/L ratios, and p-values from Welch's t-tests.

### Statistical Analysis

Unless otherwise noted, data are expressed as the mean ± S.E., and p-values were calculated using unpaired Student's t-test. Data with p-values<0.05 were determined to be statistically significant.

## Supporting Information

Table S1 **List of proteins in A549 cells identified by mass spectrometry.** Data show the IPI (International Protein Index) number, the H/L (heavy/light) ratio, the H/L variance, and the log2 spectral area for each protein, before and after quantile normalization. Additionally, the differences in H/L ratios from cells on stiff gels and cells on soft gels following quantile normalization are listed.
(XLS)

Table S2 **List of proteins in mPanc96 cells identified by mass spectrometry.** Data show the IPI (International Protein Index) number, the H/L (heavy/light) ratio, the H/L variance, and the log2 spectral area for each protein, before and after quantile normalization. Additionally, the differences in H/L ratios from cells on stiff gels and cells on soft gels following quantile normalization are listed.
(XLS)

Table S3 **List of proteins in A549 cells with statistically significant (p<0.05) differences in H/L ratios following quantile normalization.**
(XLS)

Table S4 **List of proteins in mPanc96 cells with statistically significant (p<0.05) differences in H/L ratios following quantile normalization.**
(XLS)

## References

[1] Friedl P, Alexander S (2011). Cancer invasion and the microenvironment: plasticity and reciprocity.. Cell.

[2] Paez D, Labonte MJ, Bohanes P, Zhang W, Benhanim L (2012). Cancer dormancy: a model of early dissemination and late cancer recurrence.. Clinical Cancer Research.

[3] Huang S, Ingber DE (2005). Cell tension, matrix mechanics, and cancer development.. Cancer Cell.

[4] Levental KR, Yu H, Kass L, Lakins JN, Egeblad M (2009). Matrix crosslinking forces tumor progression by enhancing integrin signaling.. Cell.

[5] Tilghman RW, Cowan CR, Mih JD, Koryakina Y, Gioeli D (2010). Matrix rigidity regulates cancer cell growth and cellular phenotype.. PLoS One.

[6] Mih JD, Sharif AS, Liu F, Marinkovic A, Symer MM (2011). A multiwell platform for studying stiffness-dependent cell biology.. PLoS ONE.

[7] Leight JL, Wozniak MA, Chen S, Lynch ML, Chen CS (2012). Matrix rigidity regulates a switch between TGF-beta1-induced apoptosis and epithelial-mesenchymal transition.. Mol Biol Cell.

[8] Barkan D, El Touny LH, Michalowski AM, Smith JA, Chu I (2010). Metastatic growth from dormant cells induced by a col-I-enriched fibrotic environment.. Cancer research.

[9] Barkan D, Green JE, Chambers AF (2010). Extracellular matrix: a gatekeeper in the transition from dormancy to metastatic growth.. European Journal of Cancer.

[10] Selbach M, Schwanhausser B, Thierfelder N, Fang Z, Khanin R (2008). Widespread changes in protein synthesis induced by microRNAs.. Nature.

[11] Sherr CJ, Roberts JM (1995). Inhibitors of mammalian G1 cyclin-dependent kinases.. Genes & Development.

[12] Klein EA, Yin L, Kothapalli D, Castagnino P, Byfield FJ (2009). Cell-cycle control by physiological matrix elasticity and in vivo tissue stiffening.. Current Biology : CB.

[13] Terry NH, White RA (2006). Flow cytometry after bromodeoxyuridine labeling to measure S and G2+M phase durations plus doubling times in vitro and in vivo.. Nat Protoc.

[14] Kostic A, Lynch CD, Sheetz MP (2009). Differential matrix rigidity response in breast cancer cell lines correlates with the tissue tropism.. PloS ONE.

[15] Aguirre-Ghiso JA (2007). Models, mechanisms and clinical evidence for cancer dormancy.. Nat Rev Cancer.

[16] Goss PE, Chambers AF (2010). Does tumour dormancy offer a therapeutic target?. Nature reviews Cancer.

[17] Schardt JA, Meyer M, Hartmann CH, Schubert F, Schmidt-Kittler O (2005). Genomic analysis of single cytokeratin-positive cells from bone marrow reveals early mutational events in breast cancer.. Cancer Cell.

[18] Uhr JW, Pantel K (2011). Controversies in clinical cancer dormancy.. Proc Natl Acad Sci USA.

[19] Barkan D, El Touny LH, Michalowski AM, Smith JA, Chu I (2010). Metastatic growth from dormant cells induced by a col-I-enriched fibrotic environment.. Cancer Res.

[20] Barkan D, Kleinman H, Simmons JL, Asmussen H, Kamaraju AK (2008). Inhibition of metastatic outgrowth from single dormant tumor cells by targeting the cytoskeleton.. Cancer Res.

[21] Kleinman HK, Martin GR (2005). Matrigel: basement membrane matrix with biological activity.. Semin Cancer Biol.

[22] Hughes CS, Postovit LM, Lajoie GA (2010). Matrigel: a complex protein mixture required for optimal growth of cell culture.. Proteomics.

[23] Soofi SS, Last JA, Liliensiek SJ, Nealey PF, Murphy CJ (2009). The elastic modulus of Matrigel as determined by atomic force microscopy.. J Struct Biol.

[24] Paszek MJ, Zahir N, Johnson KR, Lakins JN, Rozenberg GI (2005). Tensional homeostasis and the malignant phenotype.. Cancer Cell.

[25] Schrader J, Gordon-Walker TT, Aucott RL, van Deemter M, Quaas A (2011). Matrix stiffness modulates proliferation, chemotherapeutic response, and dormancy in hepatocellular carcinoma cells.. Hepatology.

[26] Sunyer R, Trepat X, Fredberg JJ, Farre R, Navajas D (2009). The temperature dependence of cell mechanics measured by atomic force microscopy.. Phys Biol.

[27] Butcher DT, Alliston T, Weaver VM (2009). A tense situation: forcing tumour progression.. Nature Reviews Cancer.

[28] Rabinowitz JD, White E (2010). Autophagy and metabolism.. Science.

[29] Benecke BJ, Ben-Ze'ev A, Penman S (1978). The control of mRNA production, translation and turnover in suspended and reattached anchorage-dependent fibroblasts.. Cell.

[30] Farmer SR, Ben-Ze'av A, Benecke BJ, Penman S (1978). Altered translatability of messenger RNA from suspended anchorage-dependent fibroblasts: reversal upon cell attachment to a surface.. Cell.

[31] Ben-Ze'ev A, Farmer SR, Penman S (1980). Protein synthesis requires cell-surface contact while nuclear events respond to cell shape in anchorage-dependent fibroblasts.. Cell.

[32] Van Schaftingen E, Jett MF, Hue L, Hers HG (1981). Control of liver 6-phosphofructokinase by fructose 2,6-bisphosphate and other effectors.. Proc Natl Acad Sci USA.

[33] Moon JS, Kim HE, Koh E, Park SH, Jin WJ (2011). Kruppel-like factor 4 (KLF4) activates the transcription of the gene for the platelet isoform of phosphofructokinase (PFKP) in breast cancer.. J Biol Chem.

[34] Gehnrich SC, Gekakis N, Sul HS (1988). Liver (B-type) phosphofructokinase mRNA. Cloning, structure, and expression.. J Biol Chem.

[35] Dunaway GA, Leung GL, Thrasher JR, Cooper MD (1978). Turnover of hepatic phosphofructokinase in normal and diabetic rats. Role of insulin and peptide stabilizing factor.. J Biol Chem.

[36] Penning TM, Drury JE (2007). Human aldo-keto reductases: Function, gene regulation, and single nucleotide polymorphisms.. Archives of Biochemistry and Biophysics.

[37] Szatrowski TP, Nathan CF (1991). Production of large amounts of hydrogen peroxide by human tumor cells.. Cancer Res.

[38] Trachootham D, Zhou Y, Zhang H, Demizu Y, Chen Z (2006). Selective killing of oncogenically transformed cells through a ROS-mediated mechanism by beta-phenylethyl isothiocyanate.. Cancer Cell.

[39] Rongvaux A, Andris F, Van Gool F, Leo O (2003). Reconstructing eukaryotic NAD metabolism.. Bioessays.

[40] Raj L, Ide T, Gurkar AU, Foley M, Schenone M (2011). Selective killing of cancer cells by a small molecule targeting the stress response to ROS.. Nature.

[41] Grigera PR, Ma L, Borgman CA, Pinto AF, Sherman NE (2012). Mass spectrometric analysis identifies a cortactin-RCC2/TD60 interaction in mitotic cells.. J Proteomics.

